# A modern-day litmus test for the sustainability of daily IVF practice - Alabama supreme court ruling overreaches in asserting that frozen embryos are legally children

**DOI:** 10.1186/s12958-024-01201-1

**Published:** 2024-03-21

**Authors:** David B. Seifer

**Affiliations:** Yale School of Medicine, 333 Cedar Street, New Haven, Connecticut 06510 USA

Since June 2022 when the Jackson v Dobbs Supreme Court federal ruling repealed the decision of Roe v Wade, we have lived in a post Roe-America fearing that it was only a matter of time before woman’s reproductive rights would be challenged one state at a time. The immediate legal challenge following the repeal of Roe vs. Wade was the challenge of women obtaining post-coital (i.e. emergency) contraception either in person or on-line. Then on February 16, 2024 an additional legal challenge was plainly directed at the daily practice of IVF which often involves discarding unused embryos that are no longer needed for treatment. The Alabama Supreme Court ruled that frozen embryos are legally children [1]. Although the court did not specifically render an opinion about a frozen embryo having “personhood” (currently defined by its supporters as life beginning at fertilization) such concern about the intentional or otherwise destruction of an embryo being construed as homicide or manslaughter of a child which could be criminally prosecuted is very troubling and daunting. For this very reason IVF clinics in Alabama went on “pause”, suspending further practice till the State Supreme Court ruling could be sorted out. Patients, physicians, nurses, embryologists, cryostorage facilities, infertility clinics, hospitals could now be vulnerable to liability and possible criminal prosecution if a frozen embryo was injured or destroyed in the course of the normal carrying out an IVF cycle. Many were “shocked” by this radical legal decision when IVF became the apparent successful target of the “personhood” movement. However, all of us should have anticipated such an eventual attempt in the aftermath of the Jackson v Dobbs ruling [2]. Since 2022 there were at least 14 states that had personhood bills at some stage of the state legislative process but none had been successfully applied to an actual case. It was never a question of whether or not a state legal challenge would come but only a question of when.

## Facts of the ruling

The Alabama Supreme Court in an 8 to 1 decision cited the state’s constitution and an 1872 statute as a basis for its ruling. Religion, as well, seems to have heavily influenced its ruling. The court wrote “human life cannot be wrongly destroyed without incurring the wrath of a holy God, who views the destruction of His image as an affront to Himself….”.

The case involved three families that each had had two children from previous successful IVF cycles and had extra frozen embryos stored at a hospital-based IVF clinic in Alabama. In December of 2020 an unauthorized psychiatric inpatient walked into an unsecured area of the clinic, opened the freezer and pulled out the storage container with the embryos but when suffering an immediate freezer burn dropped the container resulting in the destruction of the embryos. A trial court initially dismissed the three couples’ claims finding that the frozen embryos did not meet the definition of a person or a child. However, this decision was reversed by the state supreme court in what is now a landmark case that supports the theory that the loss of such frozen embryos was the legal equivalent of killing the parents’ children. The majority opinion further stated that “the wrongful death of a minor act applies to all unborn children regardless of their location including unborn children who are located outside the biological uterus at the time they are killed”.

## Possible implications of the ruling- many unanswered questions with serious consequences

The implications of this ruling have generated much fear and chaos for anyone participating in any aspect of the clinical practice of IVF, raising many more questions than answers. Are doctors, nurses, embryologists who handle embryos in the course of their daily work effectively handling live children? Are personnel and facilities (i.e. hospitals, clinics, cryo facilities) responsible and liable for destroyed embryos due to unforeseen power storages or embryos not surviving the normal thawing process? Are couples who separate (i.e. divorce) no longer disputing embryos as property but now arguing as custodians of children? Will preimplantation genetic testing now be viewed as added risk and personal liability of potentially destroying a life if the embryo were inadvertently damaged, a known and accepted risk, of such testing? Are risks of multiple pregnancies more likely to occur due to the tendency to transfer more than a single embryo at a time because freezing the extra embryo(s) may increase liability for all involved if accidentally harmed or destroyed? Would the increases in malpractice and liability insurance under this new ruling drive the cost of an IVF cycle beyond the means of most patients and result in even more restricted access to what could become an unaffordable treatment as well as make malpractice unaffordable for low volume fertility clinics? Is treatment of an ectopic pregnancy considered to be illegal by this ruling?

## Unique issue with the ruling - theocracy imbedded in a legal decision

This ruling has implications beyond just the practice of IVF. It is a threat to secular democracy. The blend of theocracy and law without separation of church and state is clearly unconstitutional. The basis of the Alabama decision breeches the First Amendment of the US Constitution and therefore, represents a severe overreach of its authority on part of the state supreme court.

## Reaction to the ruling

In less than a week following the ruling local and national politicians from both parties heard from their vocal constituents and then decried the ruling making very public their declarations to exempt IVF by carving out IVF from the scope of this new state ruling. Prior to the politicians’ outcry it was feared that such a ruling could serve as a template for other states throughout the US. It is now clearly understood by most that the US body politic overwhelmingly supports the use of IVF to build families and that this amazing 40 + year treatment is currently responsible for 2% of all births in the US.

## A prediction – going forward

As uncomfortable and threatening as this radical legal ruling is at present it is likely to serve as a litmus test as to attest to IVF’s sustainability into the future. Although it may be premature to know for sure, the reaction to this ruling highlights that it is likely that this will not prevent or materially impact the daily practice of IVF in the state of Alabama or any other state in the US in the long run. At the end of the day, as Monica Hesse wrote in the Washington Post, “embryos are vessels of hope, pain and love but they are not children.” IVF will continue legally as it is an essential therapy for the treatment of infertility and in making possible the formation of a family for those with little other choice. No state or national government is likely to obstruct its use, as family building is vital to any nation that realizes it needs a growing population for economic health and prosperity to support the demographics of its aging society. Put another way, this case and its ruling was clearly an overreach of its authority and upon its intended target. Engagement with advocacy and the political process remain as essential as ever. More assaults on women’s reproductive rights are going to follow. The next anticipated legal challenge is anyone’s guess but likely obstructed access to oral contraceptive pills and intrauterine devices will be attempted. Let’s only hope that the body politic is as quick to be assertive in providing negative feedback to their local and national politicians as they were this time. We can make this more likely if we as clinicians, scientists and educators make the effort to educate the public about reproductive issues so that future decisions are made by the people through the legislative process and not the courts, through the judicial branch.

## References

[1] https://publicportal-api.alappeals.gov/courts/68f021c4-6a44-4735-9a76-5360b2e8af13/cms/case/c93db586-ec08-4f14-a6ba-a149967e68b0/docketentrydocuments/bb88f2bf-19ca-498f-9fe2-f754d36c0ff2

[2] Letterie G, Fox D (2023). Legal personhood and frozen embryos: implications for fertility patients and providers in post-*Roe* America. J Law Biosci.

